# Asymmetric flow field-flow fractionation coupled to surface plasmon resonance detection for analysis of therapeutic proteins in blood serum

**DOI:** 10.1007/s00216-020-03011-x

**Published:** 2020-10-24

**Authors:** Mats Leeman, Willem M. Albers, Radoslaw Bombera, Johana Kuncova-Kallio, Jussipekka Tuppurainen, Lars Nilsson

**Affiliations:** 1 SOLVE Research and Consultancy AB, Medicon village, 22381 Lund, Sweden; 2 BioNavis Ltd, Hermiankatu 6-8H, 337 20 Tampere, Finland; 3 Department of Food Technology, Engineering and Nutrition, Faculty of Engineering LTH, Lund University, 22100 Lund, Sweden

**Keywords:** Multi-parametric surface plasmon resonance, Kinetics, Asymmetrical flow field-flow fractionation, Antibody, Aggregate, Detection, Binding capacity, Ligand immobilization

## Abstract

Coupling of surface plasmon resonance (SPR) detection to asymmetric flow field-flow fractionation (AF4) offers the possibility to study active fractions of bio-separations on real samples, such as serum and saliva, including the assessment of activity of possibly aggregated species. The coupling of SPR with AF4 requires the possibility to select fractions from a fractogram and redirect them to the SPR. The combination of SPR with chromatography-like methods also requires a mechanism for regeneration of the receptor immobilised onto the SPR sensor surface. In recent work, the combination of size exclusion chromatography (SEC) with SPR was pioneered as a successful methodology for identification, characterisation and quantification of active biocomponents in biological samples. In this study, the approach using AF4 is evaluated for the antibody trastuzumab in buffer and serum. The particular object of this study was to test the feasibility of using AF4 in combination with SPR to detect and quantify proteins and aggregates in complex samples such as blood serum. Also, in the investigation, three different immobilisation methods for the receptor HER-2 were compared, which involved (1) direct binding via EDC/NHS, the standard approach; (2) immobilisation via NTA-Ni-Histag complexation; and (3) biotin/avidin-linked chemistry using a regenerable form of avidin. The highest specific activity was obtained for the biotin-avidin method, while the lowest specific activity was observed for the NTA-Ni-Histag linkage. The data show that AF4 can separate trastuzumab monomers and aggregates in blood serum and that SPR has the ability to selectively monitor the elution. This is an encouraging result for automated analysis of complex biological samples using AF4-SPR.

## Introduction

As previously reported, the size separation methodology of asymmetrical flow field-flow fractionation (AF4) [1, 2] can separate the macromolecular components in whole blood, serum and plasma samples by hydrodynamic size without any major sample preparation, apart from diluting the samples [3]. Earlier studies utilizing field-flow fractionation have been reported which, in particular, focused on the larger protein assemblies (i.e. lipoprotein particles) present in serum or plasma [4–7]. Blood samples are very complex [8] containing a huge number of proteins, antibodies and other macromolecules across a very wide range of sizes [9] making quantification of a specific protein and its size distribution difficult. Size separation methods, such as size exclusion chromatography (SEC) and AF4, do not have the resolution to completely resolve all the thousands of compounds from each other. Therefore, non-selective detectors will measure on a mixture of co-eluting compounds and even a semi-quantitative determination becomes difficult or impossible at therapeutically relevant conditions. One solution to this problem is to use a selective detection methodology that can detect and quantify the compound of interest in a complex matrix. Fluorescent labelling of compounds is one commonly used approach [10–12]. However, introducing a fluorescent label onto the compound raises several concerns. First, it requires the compound to be labelled prior to whatever investigation is to be made. This may not always be possible or desirable, for example in a pharmacokinetics study in an animal model, the use of a fluorescently labelled compound would most likely not be desired. Secondly, a concern is that the label may change the physiochemical properties of the compound. For example, the introduction of the label may change a compound’s propensity to aggregate, or otherwise affect its interactions with other compounds. Instead, it would be more desirable to selectively measure directly on the target component without any labelling. One such methodology that has been used to great effect to study a wide range of components is surface plasmon resonance (SPR). SPR has been pioneered since the early 1980s by Pharmacia AB [13] and enabled the construction of the first integrated instrument for real-time kinetics analysis for biological interaction analysis (BIA) with high sensitivity [14]. SPR is based on the optical properties of a thin layer of a noble metal deposited onto a glass prism that is interrogated with an optical setup consisting of a laser and a detector to excite a “surface plasmon” [15]. The excitation of the “plasmon” requires a p-polarised laser beam impinging on the gold surface under a specific angle, and is in practice observed as a drop in reflected light when scanning the angle over the range in which the resonance occurs. If the setup is chosen so that light is reflected within the prism (under total internal reflection conditions, the so-called Kretschmann configuration), a characteristic SPR curve is produced that includes at a lower angle the onset of total internal reflection and at a higher angle the point of plasmon resonance. When compounds adsorb to the outer surface of the metal film, the refractive index close to the metal changes and the position of the SPR minimum notably changes, particularly if they are big molecules like proteins. The SPR minimum shifts up in angle when the refractive index increases, and downwards in angle when it decreases. Scanning the whole curve gives the additional advantage of determination of optical constants of the thin metal film and the layers adjacent to it by using a fitting algorithm [16]. While SPR is highly sensitive, it is not selective, because the response is caused by refractive index changes. Selectivity must be introduced by attaching to the metal surface a chemical “receptor” (ligand) that binds specifically only one or a few species from a mixture of compounds. It is often challenging to conclude if the obtained SPR signal originates from the target protein in its monomeric form or if it comes from a conjugated or aggregated form of the protein. If the protein monomer and different aggregated forms first could be physically separated and then measured by SPR, the data interpretation becomes more detailed.

Using AF4 for the size separation can have advantages over SEC [17, 18]. One is the ability of AF4 to separate complex samples with broad size distribution (such as blood serum and plasma) using little sample preparation. The surface area and shear forces are considerably lower (on the order of 1000–10,000 times for both area and shear force) in the open AF4 channel compared to an SEC column. This reduces the risk of causing changes in the sample due to shear forces. Also, the open channel of AF4 makes it much less prone to clogging, a concern when separating complex samples, such as blood plasma, with the potential to contain very large-sized impurities or components. Furthermore, it is common that high concentrations of salt are required in the SEC mobile phase to minimize interaction of the sample with the stationary phase, which may not be compatible with the analytes of interest if, for example it is of interest to study a compound and its interactions under native conditions or in a specific formulation. Separation of antibodies and their aggregates specifically using AF4 was successfully demonstrated almost 30 years ago [19].

The combination of AF4, with its ability to separate complex samples over a wide size range using a carrier with ionic strength and pH close to native conditions, and the SPR which can detect with high selectivity and sensitivity in complex sample matrixes may allow for tests that were previously not feasible or experimentally cumbersome and required sample preparation that may affect the analyte of interest.

In previous studies, SPR was successfully coupled to SEC for detection of active fractions of papain-digested antibodies [20] and for the detection of trastuzumab and the antibody-drug conjugate ado-trastuzumab-emtansine in close to native conditions [21]. In the latter, the SEC-SPR setup allowed separation of the aggregates and measurement of their affinity for the human epidermal growth factor receptor 2 (HER-2). However, the SPR analysis is crucially dependent on the immobilization method for HER-2, and thus, some alternative linking methods should be employed to increase the sensitivity of the SPR method. Previously, HER-2 was immobilised by covalent binding of the ligand to carboxymethyldextran matrices (CMD) by a combination of the carbodiimide EDC and N-hydroxysuccinimide (NHS), which is the standard approach. The great disadvantage of this standard method of binding of biomolecules to sensor surfaces is two-fold: some activity loss can occur of the receptor by immobilisation via amine groups, and due to the covalent binding, the sensor surface cannot be reused or regenerated [22]. One method to enable regeneration of the sensor surface is to use the nickel complexation between nitrilotriacetic acid (NTA) groups introduced on the CMD and a histidine tag (His_6_) on the protein [23]. The link can be broken by introduction of a high concentration of ethylenediaminetetraacetic acid (EDTA). This enables, in principle, also a better specific activity of the immobilised protein, because the histidine tag can be introduced by genetic engineering at the appropriate site for immobilisation. However, the method often lacks the stability of immobilisation, the ligand slowly dissociating from the surface during long experiments, and for this reason, covalent fixation has been proposed, which yet annuls the regeneration function. Biotin-avidin complexation is well-established in biotechnology applications to create biohybrid structures [24] and for obtaining very stable binding of ligands to sensor surfaces [25]. Although the basic interaction between biotin and avidin is chemically difficult to break, recently, a regenerable mutant of avidin has been manufactured that allows stable binding but also regeneration of the sensor surface [26, 27]. The idea of the method is to produce a highly stable biotinylated sensor surface, and first immobilise the avidin mutant, after which a biotinylated probe of the ligand is introduced. This yields two levels of regeneration: with a weak reagent to break the bond between the ligand and the analyte, or with a strong reagent to remove the ligand and the avidin.

In this study, we propose AF4-SPR as an orthogonal method that can be used across the whole spectrum of biological samples, and give a preliminary account of the effect of three ligand immobilisation methods on the results in terms of kinetic and equilibrium constants.

## Materials and methods

### Materials

The carrier for the AF4 was prepared from analytical grade sodium chloride (prod. no. 27788.297, VWR), di-sodium phosphate dihydrate (prod. no. 28029.292, VWR), potassium phosphate (prod. no. 26936.293, VWR), potassium chloride (prod. no. 26764.298, VWR) and sodium azide (prod. no. 103692K, VWR). For the aggregation buffer, sodium acetate (prod. no. A13184, Alfa Aesar) was used. Water used for the preparation was purified on an ion-exchange unit (Purelab Ultra, ELGA). Standard proteins myoglobin (prod. no. M1882), ovalbumin (prod. no. A5503), bovine serum albumin (prod. no. A3059), immunoglobulin G (prod. no. 56834) and thyroglobulin (prod. no. T1001) as well as the human serum (prod. no. H4522) were obtained from Sigma-Aldrich.

The trastuzumab was obtained from a Swedish pharmacy (Apoteket AB). Purified HER-2 was acquired in two modified forms from Sino Biological Inc.: His-tagged (prod. no. 10004-H08H) and biotinylated and His-tagged (prod. no. 10004-H08H-B). The molecular weight of the HER-2 protein was 71 kDa, and after reconstitution, the concentration of both stock solutions was 250 μg/mL.

Biotinylated Au sensors, NTA-modified carboxymethyldextran sensors (CMD-NTA) and unmodified carboxymethyldextran sensors (CMD) were provided by BioNavis Ltd.

### Methods

#### Inducing aggregation

Trastuzumab was obtained as a dry powder. Stock solution was prepared by dissolving the trastuzumab powder in water to an antibody concentration of 50 mg/mL. To induce aggregation, the salt concentration and pH of the trastuzumab solution was changed by diluting an aliquot of the stock solution 50 times in an aqueous solution of 20 mM sodium acetate, 50 mM sodium chloride, pH 4.6. Aggregation was then induced by heating at 75 °C for 30 min. Dynamic light scattering (DLS) was performed on the trastuzumab solution, before and after heating, to verify that aggregation occurred. The DLS measurements were performed on a Nanosizer ZS (Malvern), duplicate measurements at 173° scattering angle, measurement time of 70 s.

#### Separation by asymmetrical flow field-flow fractionation

The separations by asymmetrical flow field-flow fractionation (AF4) were performed on an Eclipse 3 unit (Wyatt technology) connected to a chromatographic system from Agilent (1100-series). The chromatograph consisted of a vacuum degasser, pump, autosampler and UV/VIS diode array detector. The UV detector monitored at a wavelength of 280 nm. The control of the AF4 and HPLC units was achieved by the Voyager software (Wyatt) while data collection was by Astra 6.1.2 (Wyatt).

The AF4 separation was performed on a SC channel (Wyatt) utilizing a 490-μm-thick and 22-mm-wide channel on top of a 10-kDa regenerated cellulose membrane. The carrier for the AF4 was phosphate-buffered saline (PBS) consisting of 137 mM sodium chloride, 10 mM disodium hydrogen phosphate, 2 mM potassium dihydrogen phosphate, 3 mM potassium chloride, pH 7.4 with 3 mM sodium azide added to prevent microbial growth. Injection volume was 20 μL. Separations were performed at ambient temperature (approximately 22 °C). The detector flow rate was 0.5 mL/min, and the crossflow rate was 2 mL/min, kept constant for 16 min, whereafter the crossflow rate was decreased exponentially according to the below equation:

$$ {Q}_c(t)={Q}_{c,0}\cdotp {2}^{-\frac{t}{t_{1/2}}} $$

where *Q*_*t* = 0_ is the volumetric crossflow rate at the onset of the decay, *t* is the time, and *t*_½_ is the decay rate (16 min in the present study). When the crossflow rate reached 0.15 mL/min, it was kept constant for the reminder of the separation.

To ensure that the AF4 unit was having sufficient resolution and that the UV-MALS and RI detectors were performing as intended, a mixture of standard proteins (myoglobin, ovalbumin, bovine serum albumin, immunoglobulin G, and thyroglobulin) was analysed.

#### Immobilization of HER-2 for MP-SPR analysis

HER-2 was immobilised in situ using the MP-SPR Navi™ 220A NAALI system (BioNavis Ltd.). The instrument configuration consisted of two flow channels and two measurement wavelengths per flow channel (670 and 785 nm). The HER-2 receptor was immobilized in one flow channel while the other one was used as a blank, having gone through all the treatments as the first channel, but lacking the HER-2 receptor.

Three different immobilization methods for HER-2 receptor were used for comparison: (1) with carboxymethyl dextran sensor (type CMD), coupling the His-tagged HER-2 protein to the sensor surface according to the standard amine coupling chemistry using EDC and NHS; (2) with CMD-NTA sensors, capturing the his-tagged HER-2 protein via nickel ion complexation; and (3) immobilizing the biotinylated and His-tagged HER-2 via “regenerable avidin” to the biotin sensor. Incubation times were typically 4 to 7 min for the ligands to reach maximum binding. For the CMD sensor, the HER-2 receptor was immobilised according to a recent publication [21]. In the case of the CMD-NTA coating, the surface was first conditioned with 25 mM NaOH and 250 mM EDTA and then activated with 5 mM NiCl_2_, followed by coupling of the His-tagged HER-2 in HBS-ET buffer (10 mM HEPES, 150 mM NaCl, 50 μM EDTA and 0.05% Tween 20, pH = 7.4 “HBS-ET”) at 50 μg/mL concentration. The immobilization protocol with the biotinylated sensors was carried out first by coupling of the regenerable avidin mutant at 25 μg/mL, and then by coupling of biotinylated His-tagged HER-2 at 25 μg/mL. These reactions were conducted in PBS buffer pH = 7.4 with 0.05% Tween 20.

#### Titration experiments with trastuzumab

Titration experiments were carried out with the abovementioned MP-SPR Navi™ instrument, using BioNavis control software version 6.4. Data processing and analysis was performed with BioNavis Viewer and TraceDrawer™ version 1.8 (Ridgeview Instruments AB, SE).

Binding of trastuzumab was performed in the low nanomolar range up to 66 nM (10 μg/mL) with regeneration of the receptor between the standards and samples, using 10 mM glycine buffer, pH = 2.0. (“multicycle titration”). The regeneration buffer, however, dissociated the HER-2 from the CMD-NTA sensor, so titrations were performed compensating for loss of receptor. Data processing with TraceDrawer™ enabled to obtain the kinetic constants *k*_a_, *k*_d_ and the dissociation constant *K*_D_. Data were analysed additionally with the “Langmuir-Freundlich” isotherm on the equilibrium binding levels, to obtain salient information about the heterogeneity and/or cooperativity of the binding [28]. This isotherm also provided a means of evaluation of the concentrations of the samples (using Hill plot option) with TraceDrawer™. The assay buffer was PBS-T (150 mM NaCl, 10 mM phosphate, pH = 7.4, 0.05% Tween 20, pH = 7.4) in case of the CMD and biotin sensors and HBS-ET in case of the CMD-NTA sensor.

## Results and discussion

### Optimisation of SPR method

#### Immobilization of HER-2 receptor

Immobilization levels of the HER-2 receptor can be evaluated from signals (in millidegrees) registered by SPR at each detection wavelength (here 670 and 785 nm). They are summarized in Table 1 and expressed as the surface density of the protein which can be inferred from the SPR response at 785 nm (1 mdeg ≈ 1 ng/cm^2^). Considering the molecular weight of the ligand HER-2 (71 kDa) and analyte molecule (150 kDa), the minimum immobilization level of HER-2 necessary for SPR kinetic measurement was estimated to 48 mdeg, i.e. 48 ng/cm^2^ [29]. With the CMD-type sensors (CMD and CMD-NTA), total binding of the ligand is expectedly higher (even up to 500 mdeg) than sensors based on monolayers (biotin sensors) due to a thick hydrogel layer. The responses registered with the biotinylated Au sensors are generally around 350 mdeg for avidin binding, and 100–200 mdeg for ligand capture depending on the protein MW. The here applied experimental protocol of HER-2 immobilization onto 3 different types of sensor provided a sufficient amount of the ligand for further kinetic measurements. Interestingly, the largest amount of HER-2 was coupled to the CMD-NTA sensor due to a high sensor capacity to complex His-tag groups from the ligand protein. However, the complex appeared to be less stable and was completely dissociated from the surface by injection the regeneration buffer: 10 mM glycine pH = 2.0 (data not shown). Such mild regeneration conditions were further applied in kinetic measurements between injections of analyte (standard at different concentrations or trastuzumab fractions from AF4) in case of HER-2 immobilized onto CMD and biotin-avidin sensors. Due to instability of NTA-Ni-His (HER-2) complex, the analyte solutions were sequentially introduced, not using the glycine regeneration in-between.

**Table 1:** Immobilization levels of the HER-2 on three different sensor surfaces

Immobilization method	Surface coverage, *Γ* (ng/cm^2^)
CMD, covalent linking	174
CMD-NTA, Ni complexation	484
Biotin-avidin binding	74

#### Kinetic and equilibrium analysis compared

**Fig. 1:**
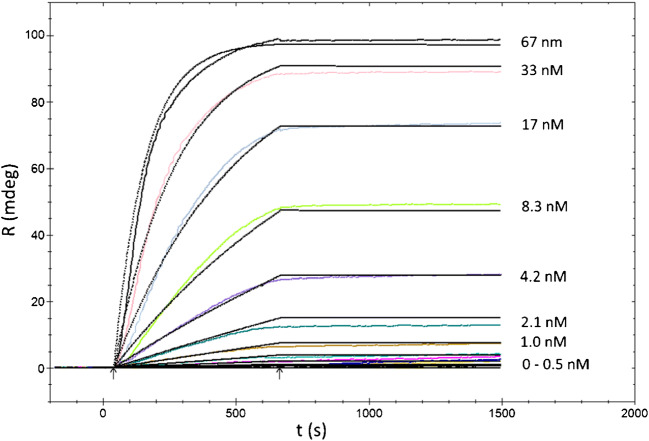
Titration of trastuzumab against the HER-2 receptor immobilized on the CMD sensor. Coloured curves represent the measured data while black solid curves follow the fitting results (chi^2^ = 1.97mdeg^2^). Kinetics analysis results along with standard errors are grouped in Table 2. Data analysis is performed using TraceDrawer™. Arrows indicate the start and end of the injection

An example of a titration experiment is presented in Fig. 1 for the case of the CMD sensor where sensograms represent subsequent injections of trastuzumab at increasing concentrations. The kinetic measurement data analysis was performed using TraceDrawer™ which results for the three immobilization methods summarised in Table 2. The obtained *K*_D_ values were of the same order on all three tested sensors and being below nanomolar proved high affinity of trastuzumab to the assayed HER-2 receptor. Interestingly, the dissociation constant was slightly lower for CMD sensor as compared to the two other surfaces which evidenced somewhat higher affinity to the analyte. This could be explained by a higher accessibility of the receptor binding sites in comparison to CMD-NTA and biotin-avidin sensors where the HER-2 was modified with His and biotin tag respectively. The calculated fitting curves were rather precise (chi^2^ below 2mdeg^2^), although the interaction was quite irreversible without any significant dissociation occurring within the given dissociation time. The absence of important dissociation demonstrated a strong binding of the antibody to the receptor which was expressed by the calculated kinetic constant values. One might notice that at low concentrations, the interaction clearly became mass transport limited as evidenced by more linear association curves.

**Table 2:** Kinetics results for the three immobilization methods of trastuzumab vs. HER-2. *R*_max_ is the maximum binding level, *k*_*a*_ and *k*_*d*_ are the association and dissociation rate constants, *K*_*D*_ is the dissociation constant and *k*_*t*_ the mass transfer coefficient

		CMD		CMD-NTA		Biotin-avidin	
Parameter	Unit	Value	Error	Value	Error	Value	Error
*R*_max_	mdeg	9.8E+01	5.7E−03	1.0E+02	4.0E−02	5.7E+01	1.3E−02
*k*_a_	M^−1^ s^−1^	1.3E+05	2.7E+02	3.5E+05	1.4E+03	3.9E+05	1.7E+02
*k*_d_	s^−1^	2.0E−06	3.1E−04	1.7E−04	4.1E−05	2.6E−05	2.9E−08
*K*_D_	M	1.5E-11	2.4E−09	4.9E−11	1.2E−10	6.5E−11	1.0E−04
*k*_t_	mdeg M^−1^ s^−1^	6.3E+08	1.2E+06	2.9E+08	3.0E+04	8.0E+08	2.4E+06

Equilibrium data analysis was performed by Hill plot function (TraceDrawer™) and confirmed with Langmuir-Freundlich isotherm which results are grouped in Table 3. The equilibrium analysis yielded more reliable data for the dissociation constant which was in nanomolar range and again evidenced high affinity of the receptor to trastuzumab. However, it also gave evidence of heterogeneity of binding and some degree of cooperativity expressed by the exponent of the Langmuir-Freundlich isotherm lying between 1 and 2. Such nature of interaction between antibody and HER-2 receptor is expected if we take into account the antibody binding to a densely immobilized, chemically modified (His and biotin tags) antigen.

**Table 3:** Equilibrium results for the three immobilization methods of trastuzumab vs. HER-2

		CMD		CMD-NTA		Biotin-avidin	
Parameter*	Unit	Value	Error	Value	Error	Value	Error
*R*_max_	mdeg	1.0E+02	8.8E−04	6.8E+01	1.3E−02	6.2E+01	1.1E−03
*K*_D_	nM	9.4E+00	6.6E−04	2.3E+00	1.1E−03	6.0E+00	7.4E−03
*n*		1.3E+00	9.6E−05	1.7E+00	1.8E−03	1.2E+00	2.4E−04

*The isotherm used was: $R={R}_{\mathrm{max}}{C}^n/\left({K}_D^n+{C}^n\right)$ where *C* is the molar concentration of trastuzumab and *n* the binding exponent (Hill slope)

### Trastuzumab aggregation

#### Inducing aggregation

**Fig. 2:**
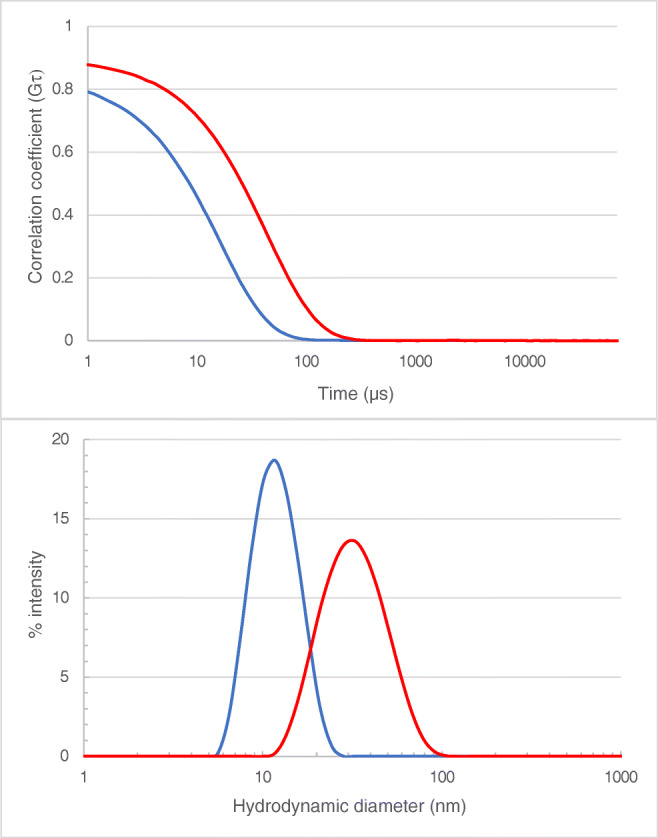
Dynamic light scattering data for trastuzumab solution, before (blue trace) and after aggregation, has been induced (red trace). The bottom graph shows the size distribution obtained from the correlation function (top graph)

Therapeutic antibodies are formulated to give stable solutions with a minimum degree of aggregation. However, in this case, it was desired to have aggregates to test if the analytical system is capable of detecting such aggregates. For that reason, trastuzumab (serving as a model representing a therapeutically relevant compound) was “stressed”. First the trastuzumab solution composition was changed by lowering the pH and changing the salt composition; thereafter, the solution was stressed by subjecting the solution to an elevated temperature of 75 °C for 30 min. To monitor the effect of the treatment as well as the stability of the solutions, it was analysed by dynamic light scattering (DLS), as depicted in Fig. 2.

The DLS correlation data clearly shows that the treatment had an effect on the size and size distribution of the trastuzumab when compared to a solution of unstressed trastuzumab. The unstressed trastuzumab gives a clean correlation graph, and the size distribution is relatively narrow with an average hydrodynamic size of 11.2 nm and a polydispersity of 0.08 which is data that can be expected for an antibody in monomeric form [30]. For the stressed solution, the size distribution was, however, significantly broader—the average size is 30 nm and a PDI of 0.125 is obtained.

#### Size separation of trastuzumab before and after inducing aggregation

**Fig. 3:**
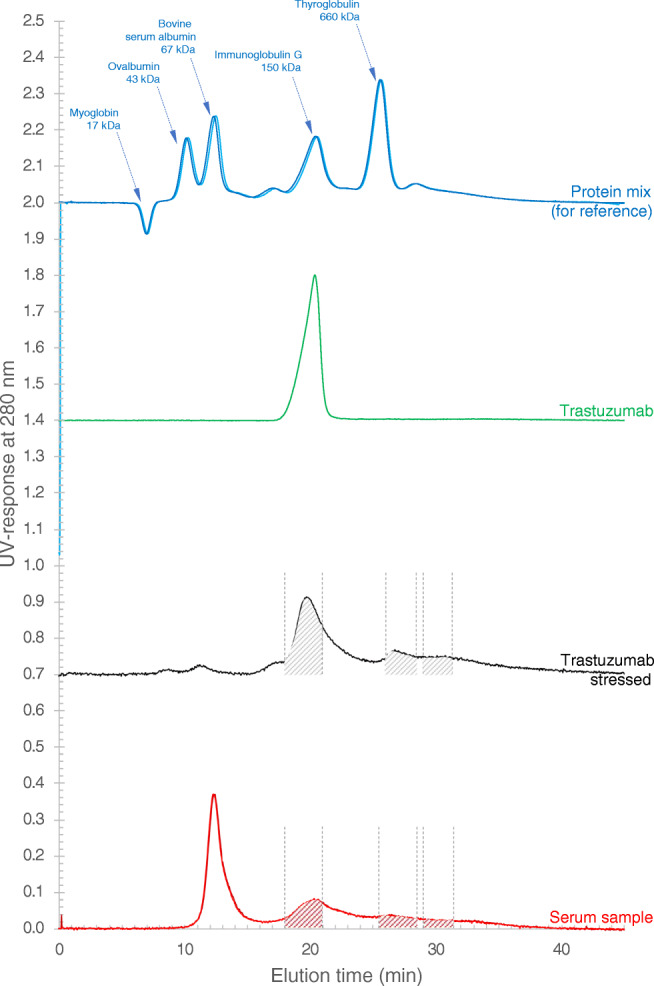
Asymmetrical flow field-flow fractionation data for trastuzumab solution, before (green trace) and after aggregation (black trace), as well as for blood serum (duplicate analysis, red traces). For reference, the trace from the duplicate analysis of a protein mix containing 17 kDa myoglobin, 43 kDa ovalbumin, 67 kDa bovine serum albumin, 150 kDa immunoglobulin G and 660 kDa thyroglobulin (blue traces) is also included. Eluted fractions monitored by UV at 280 nm. To facilitate comparison of elution times, the traces are in the same graph. Signals off-set (on y-axis) for clarity

While dynamic light scattering is a useful tool for monitoring the aggregation of protein solutions, it does not give reliable quantitative data with high resolution. To obtain an appropriately resolved understanding of the size range of the trastuzumab aggregates, the stressed solution was analysed by AF4 (black trace, Fig. 3). For reference, the unstressed solution was also analysed (see green trace in Fig. 3).

It is clear from the AF4 analysis that the unstressed trastuzumab had a well-defined peak eluting in the 17.5–22.0 min time interval (peak max at 20.5 min). There are no signs of aggregates, as expected from a well formulated antibody. The elution time of the trastuzumab also agreed well with the elution time of the IgG of the protein mixture (peak maximum at 20.5 min, see blue trace in Fig. 3).

In contrast, the stressed trastuzumab showed a much broader and multimodal elution pattern. There were components eluting earlier than the monomeric antibody (i.e. fragments)—one size population with peak maximum at 8.8 min (likely a 25 kDa fragment), a second size population with peak maximum at 11.3 min (likely a 50 kDa fragment) and finally a size population in the elution time range from 16 to 18 min (likely a 100 kDa fragment). As for the non-stressed trastuzumab, the major component was beginning to be eluted at 18 min (peak maximum at 20 min) which was assumed to be the intact monomeric species. However, for the stressed trastuzumab, the main peak was tailing up to 25 min—potentially due to partially denatured monomeric species and then dimers and other oligomeric forms.

Thereafter, several larger sized components were detected—one size population eluting from 25 to 29.5 min (peak maximum at 27 min). Comparing with the elution time of the protein mixture (blue trace), this was estimated to correspond to proteins with molar mass in the range from 500 to 1000 kDa. Finally, there was a broad peak from 29.5 to 40 min representing larger aggregates estimated to have a molar mass in excess of 1 MDa.

It should be noted that the different components of the stressed trastuzumab are only partially resolved, and therefore, there was likely co-elution of different aggregated forms. The molar mass was estimated based on comparison with the elution time of the standard proteins. The tentative identification and molar mass estimates of the early eluted components (8–18 min) were based on the commonly observed fragmentation pattern of antibodies into 25, 50 and 100 kDa fragments as well as the comparison with the elution time of the standard proteins in the 17–150 kDa range.

### Trastuzumab analysis in serum

#### Size separation of stressed trastuzumab in serum

As reported earlier [3], blood serum samples may be size separated and analysed on AF4 without any special sample pre-treatment. However, a dilution step is needed to reduce the viscosity of the serum and ensure compatibility and proper function of the autosampler. The UV detector trace from the analysis of a serum sample is included in Fig. 3 which shows the typical elution pattern for blood serum, that is—the most abundant component—serum albumin peak max at 12.4 min, the slightly less abundant IgG at 20.5 min and then larger blood serum components continuously eluting from the channel.

To investigate the possibility to use SPR to detect trastuzumab (and aggregated forms of trastuzumab), the serum sample were spiked with stressed trastuzumab for a concentration of 500 μg stressed trastuzumab/mL serum. The spiked serum was incubated 30 min at room temperature before diluting 100 times with PBS and then performing the size separation by AF4.

Upon size separation, 3 fractions were collected: fraction 1—components eluting from 18.0 to 21.0 min (monomers expected); fraction 2—components eluting from 26.5 to 29.5 min (presumably oligomers); and finally, fraction 3—components eluting from 30.0 to 33.0 min (presumably larger aggregates). To allow comparison, fractions were also collected from the AF4 separation of stressed trastuzumab without serum components (i.e., in PBS) at the same time intervals (the fraction 1 represents approximately 33% of the eluted trastuzumab in PBS).

The stressed trastuzumab when spiked into the serum could not be detected by UV for two reasons. Mainly because the concentration was (after the dilution) too low. Secondly, because the presence of the native serum proteins (present at significantly higher concentration than trastuzumab) would be co-eluting with the trastuzumab species and thereby interfered with the direct detection of the trastuzumab.

#### Quantification of trastuzumab fractions by MP-SPR

**Fig. 4:**
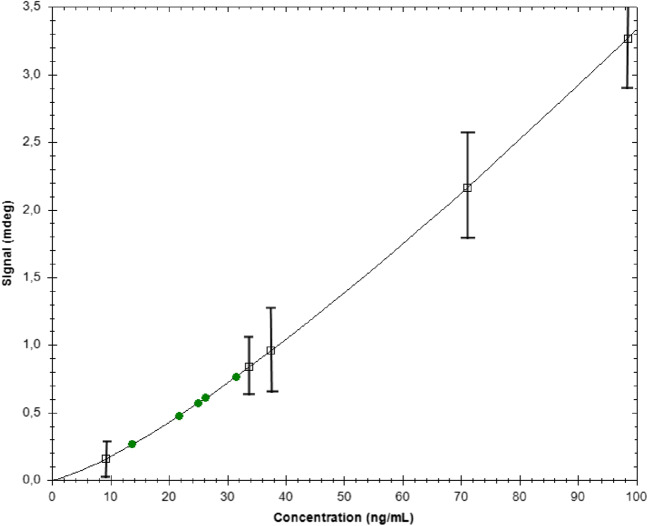
Calibration plot for trastuzumab standard samples (10 ng/mL–100 ng/mL) on the CMD sensor (chi^2^ = 0.54mdeg^2^). Squares indicate the standards and green markers the assayed AF4-collected fractions. Data analysis is provided by TraceDrawer™ with results summarized in Tables 3 and 4

To circumvent the non-selective UV detection, MP-SPR was used to quantify trastuzumab in the fractions collected from the AF4. Calibration curves were made by replotting the equilibrium data in *ng/mL* concentration scale and evaluating the concentrations with the TraceDrawer™ software in the case of the CMD sensor. Samples were not assayed using the CMD-NTA sensor due to lower stability of the HER-2 protein onto the surface (protein dissociation). Neither the biotin-avidin surface was applied because of the low binding capacity for the receptor (74 mdeg ≈ 74 ng/cm^2^, see Table 1). The calibration curve was fitted to the “four parameter equilibrium Lo-Hi function”, which is also known as a “Hill Plot”. This function is mathematically fully equivalent to the Langmuir-Freundlich isotherm. The samples fell in the lower part of the calibration curve, just above the lowest standard (Fig. 4). The obtained values are given in Table 4.

**Table 4:** Analysis results for the TZMab samples

Matrix	Fraction	*R* (mdeg)	C (ng/mL)
PBS	F1	0.761	31
	F2	0.604	26
	F3	0.255	13
SERUM	F1	0.579	25
	F2	0.470	22
	F3	− 0.070	<DL

The data show that both monomers, oligomers and higher aggregates of trastuzumab could be detected and quantified by SPR from the AF4 fractions of stressed trastuzumab in PBS. Similarly, for the stressed trastuzumab spiked into blood serum, the monomer and oligomer fractions were successfully detected, although the estimated concentration is 15–19% lower than obtained for trastuzumab in PBS. However, the large trastuzumab aggregates (eluted in fraction 3) were not detected when present in a serum matrix. Since the aggregates are detected by SPR when analysed in pure in PBS, this may be an indication that the large aggregates are interacting with some of the components in the blood serum matrix, making the large trastuzumab aggregates inaccessible to the SPR sensor surface.

## Conclusions

In this study, we successfully assessed the possibility to use SPR as a detection and characterization tool for compounds which were size fractionated with AF4. It is shown that an antibody and its aggregates (size separated and eluted from the AF4) can be detected by the SPR, not only in simple buffer systems but also when the antibody was in the more complex matrixes of blood serum. Thus, this technology may be used to gain insight into how proteins, antibodies and other biomolecules behave in the complex matrices, such as the blood system of animals and humans. This is also of interest when studying the interactions and stability of, for example proteins and antibodies in such complex media. Although the results presented here reflect analysis in blood, the methodology is likely to be extendable to other body fluids as well.

With respect to the immobilization methods for HER-2, the critical factor is always the final amount of active binding sites available on the sensor surface. The optimisation of the immobilisation efficiency will always depend on the initial specific activity of the protein preparation and the loss of binding sites due to immobilisation. In this study, it was observed that the largest number of binding sites was still provided by covalent immobilisation on CMD, yielding possibilities for assessment of trastuzumab concentrations in buffer and serum. However, when considering the amount of available binding sites relative to the total amount of HER-2 linked to the sensor surface (compare the amounts immobilised in Table 1 with the *R*_max_ values of Table 3), the avidin-biotin method was clearly more efficient, and the CMD-NTA method clearly least efficient.

The present study represents a proof-of-concept approach which can be ultimately developed towards automated online measurements. Coupling the SPR detector directly to the AF4 system requires some further procedural and methodological developments, which we hope to report in the near future.
